# Decellularised extracellular matrix-based biomaterials for repair and regeneration of central nervous system

**DOI:** 10.1017/erm.2021.22

**Published:** 2022-01-07

**Authors:** Burcu Yaldiz, Pelin Saglam-Metiner, Ozlem Yesil-Celiktas

**Affiliations:** Department of Bioengineering, Faculty of Engineering, Ege University, 35100 Izmir, Turkey

**Keywords:** Brain decellularisation, central nervous system, extracellular matrix, spinal cord decellularisation, stem cells

## Abstract

The central nervous system (CNS), consisting of the brain and spinal cord, regulates the mind and functions of the organs. CNS diseases, leading to changes in neurological functions in corresponding sites and causing long-term disability, represent one of the major public health issues with significant clinical and economic burdens worldwide. In particular, the abnormal changes in the extracellular matrix under various disease conditions have been demonstrated as one of the main factors that can alter normal cell function and reduce the neuroregeneration potential in damaged tissue. Decellularised extracellular matrix (dECM)-based biomaterials have been recently utilised for CNS applications, closely mimicking the native tissue. dECM retains tissue-specific components, including proteoglycan as well as structural and functional proteins. Due to their unique composition, these biomaterials can stimulate sensitive repair mechanisms associated with CNS damages. Herein, we discuss the decellularisation of the brain and spinal cord as well as recellularisation of acellular matrix and the recent progress in the utilisation of brain and spinal cord dECM.

## Introduction

The human central nervous system (CNS) is a highly complex biological tissue, comprised of neurons and support cells called glia (Refs 1, 2). Together with neurons, glial cells produce and assemble a highly organised extracellular matrix (ECM) that makes up approximately 20% of the total volume of the adult CNS (Refs 3, 4, 5). The ECM has a unique composition and is mainly composed of proteoglycans (aggrecan, brevican, neurocan and versican), glycosaminoglycans (GAGs) (e.g., hyaluronan), glycoproteins (e.g., tenascin-R and tenascin-C), and relatively low levels of fibrous proteins such as collagen and fibronectin (Refs 6, 7). Today, it is known that each ECM component in the CNS plays diverse roles in neurite outgrowth, axonal guidance, cell migration and differentiation as well as synaptogenesis (Refs 8, 9).

Once formed, the composition of the mature ECM is relatively stable under physiological conditions (Refs 10, 11). However, inflammatory responses to prevent the expansion of tissue damage after traumatic injuries or during neurodegenerative diseases alter the composition of the CNS ECM and result in remodelling (Ref. 12). Tissue damage leads to upregulation and overexpression of some inhibitors along with a breakdown of ECM molecules, altering ECM homeostasis and the ability to prevent neurodegeneration (Refs 13, 14, 15, 16). Although neurogenesis and migration are increased to the injured area, most of the new neurons die or migrate to other areas because of the improper environment (Ref. 17).

Over the past decade, numerous studies have revealed that biomaterials have promoted CNS tissue regeneration (Refs 18, 19, 20) through enhancing cell-tissue attachment and/or entrapment by mimicking the native tissue, as well as improving the environment to provide vascularisation and promote cell survival (Refs 21, 22). Although individual ECM components, such as collagen, hyaluronic acid, and fibronectin scaffolds, have been developed for CNS applications, the majority of the solutions have not fully recapitulated the native tissue due to the highly complex composition of the ECM. Therefore, decellularised CNS ECM recently utilised as the native tissue can be more closely mimicked.

Decellularisation is the process of removing all cellular and nuclear components from a tissue or an organ to prevent an initial immune response while preserving the ultrastructure and composition of the native ECM (Refs 23, 24). The decellularised tissue can be in the form of a porous solid, ground to obtain powder form and gelated to formulate a hydrogel. The well-preserved decellularised ECM possesses similar concentrations and ratios of GAGs, fibrous and adhesion proteins and can promote the regenerative response specific to the ECM's tissue or organ of origin (Ref. 25). Furthermore, decellularised extracellular matrix (dECM) can be well tolerated even by xenogeneic recipients as the molecules are conserved across species (Ref. 26). Compared to solid dECM, injectable form is more suitable for CNS applications, particularly for neurogeneration, allowing a minimally invasive delivery without the need of surgical access for implantation (Ref. 27).

For effective neuroregeneration, stem cells (SCs) with responses such as mitogenesis, chemotaxis and secretion of several neurotrophic factors with subsequent neuroprotective/neurorestorative effects have been reported (Refs 28, 29). Transplanted SCs have to survive, grow, proliferate and differentiate specific to its location and also integrate into the host circulatory system for accomplishing regenerative outcomes (Refs 30, 31). For recellularisation studies of brain dECM scaffolds and neurorestoration via cell transplantation, mesenchymal SCs (MSCs) and induced pluripotent SCs (iPSCs) have been utilised (Ref. 29). MSCs are widely studied adult SCs as part of therapeutic cell transplantation to repair failed tissues and organs for regenerative medicine and treatment of various diseases such as Parkinson's, and ischemic stroke with the capability to replace and regenerate damaged tissues with no immunogenic effect (Refs 32, 33, 34). Various studies have shown that neural, bone marrow and adipose-derived MSCs can be induced to express a neuronal cell phenotype, trans/differentiate to neural precursors and/or mature neurons and promote neuroprotection and neurogenesis *in vitro* under specific experimental conditions for the treatment of CNS diseases (Refs 28, 35, 36, 37, 38). On the other hand, iPSCs are embryonic SC-like cells that are reprogrammed from somatic cells to a pluripotent stage by the induction of specific transcription factors (four well-described reprogramming factors: Oct4, Sox2, c-Myc and Klf4), having the capacity of unlimited self-renewal and differentiation to create all three germ lines and all cell types in the human body, including neurons (Ref. 39). The immunogenicity of iPSCs has been reported to change due to fatal errors or mutations during reprogramming and differentiation processes, while the risk of rejection has been shown to be low when administered autologously (Ref. 33), also presenting a potential as a regenerative medicine in CNS diseases (Refs 29, 40).

**Fig. 1.**
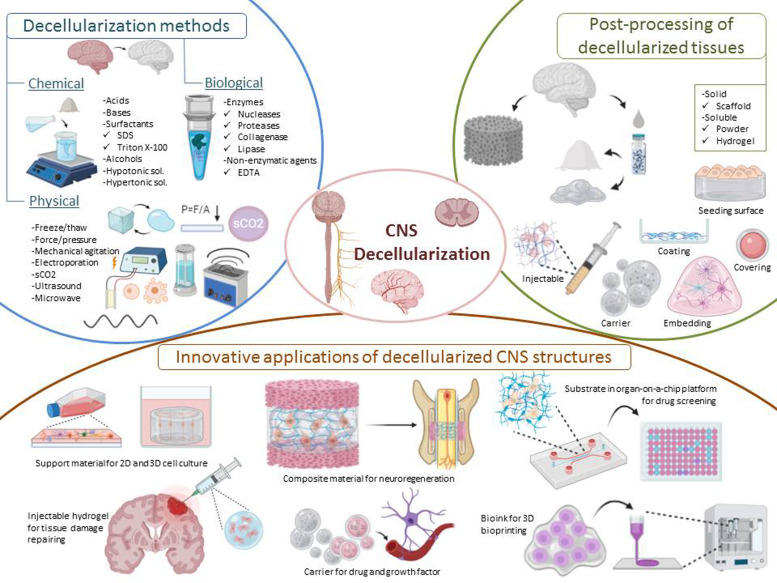
Representative image of decellularisation methods, post-processing of decellularised tissues and innovative applications of CNS structures.

Apart from highlighted applications, dECM-based biomaterials have been utilised in various tissue engineering approaches such as support materials for 3D cell culture studies (e.g., brain organoids), injectable hydrogels for the repair of tissue damage, carriers for drug and growth factors, composite materials for neuroregeneration, substrates mimicking native tissue microenvironment in organ-on-a-chip platforms, and bioinks for 3D bioprinting (Fig. 1). This review focuses on the physical, chemical and biological methods for decellularisation of brain and spinal cord ECM in the context of CNS applications and specifically discusses innovative applications.

## Decellularisation methods

As the decellularisation process requires a delicate balance between the preservation of native ECM structure and effective cell removal, complete removal of all cellular components such as DNA, nuclei, membrane lipids from a tissue is not possible. However, these cellular remnant components have been shown to initiate a pro-inflammatory response upon *in vivo* implantation, resulting in inhibition of constructive tissue remodelling. Therefore, three criteria have been proposed to prevent an adverse host response; (i) the amount of DNA should be <50 ng/mg dry weight of ECM, (ii) the length of DNA fragments should be <200 bp, and (3) visible nuclear material, cell debris, endotoxin and bacterial contamination should not be observed in histological analysis (Ref. 41). However, DNA fragments have to be <24 bp for CNS applications as immune system cells such as microglia and mast cells can recognise DNA fragments of 24 bp. Residual DNA, degraded ECM components and cell debris in the decellularised tissue turn into a damage-associated molecular pattern (DAMP) when break down, leading to unwanted inflammation activation through Toll-like receptors, microRNA activation and secretion of IL-6, IL-1, TNF and IFN-*γ* as pro-inflammatory cytokines in M1 macrophage polarisation. Although some of the DAMPs have constructive functions, often destructive effects are observed on ECM (Refs 42, 43, 44).

Decellularisation protocol depends upon several factors such as cell density, lipid content and thickness of the tissue (Ref. 45), which have to be taken into account for an effective decellularisation. In this context, various methods including physical, chemical and biological methods have been employed.

The majority of the physical methods rely on heating, cooling, mechanical and electrical approaches (Table 1). Freeze–thaw consists of freezing tissues or organs at −80 °C and thawing it at biological temperature (37 °C). During the process, intracellular ice crystal formation disrupts cell membranes, leading to cell lysis. However, multiple freeze–thaw cycles or the combination of freeze–thaw cycles and detergents may be required to enhance decellularisation, especially in dense tissues (Ref. 46). It is important to note that freeze–thaw may cause certain disruption of the ECM ultrastructure (Ref. 47). Cells on the surface of a thin tissue organ (i.e., small intestine and skin) can be effectively removed by mechanical abrasion (Ref. 48). However, this method is often used by coupling with hypertonic saline, enzymes, or chelating agents to facilitate efficient detachment of cells from underlying basement membrane (Ref. 49).

**Table 1.** A summary of physical methods

Agents/techniques	Mode of action	Advantage/disadvantage	Tissue/organ	Ref.
Freeze/thaw	Disrupts cell membrane through formation of ice crystals	Can disrupt the ECM ultrastructure Requires multiple cycles	Liver, tendon	(Refs 46, 47)
Mechanical abrasion	Removes cells on the surface by applying mechanical force	Requires chemical and/or biological agents to facilitate detachment of cells	Amniotic membrane	(Ref. 48)
Mechanical agitation, sonication	Enhances decellularisation	Can cause the disruption of ECM structure Enhances the penetration of agents into tissue Facilitates the removal of cellular components	Meniscus	(Ref. 53)
Supercritical fluids	Applies CO_2_ at pressures and temperatures above 7.40 MPa and 31 °C	Requires entrainer to remove charged molecules Maintains ECM structure Rapid decellularisation Enhances protein stability	Cornea, aorta	(Refs 55, 60)
Non-thermal irreversible electroporation	Causes the formation of micropores in the cell membrane	Maintains the tissue scaffold and large blood vessels	Carotid artery, liver	(Refs 51, 52)

In the non-thermal irreversible electroporation process, microsecond electrical pulses induce the formation of irreversible micropores in the cell membrane lipid bilayer, causing cell death through loss of cell homeostasis, while preserving the tissue scaffold and large blood vessels (Refs 50, 51, 52). Mechanical agitation and sonication are other approaches used in combination with chemical or biological decellularisation agents as the penetration of agents into tissue is enhanced and the removal of cellular components is facilitated by improving mass transfer in the system (Refs 24, 53). In addition to conventional physical methods, supercritical carbon dioxide (SC-CO_2_) has been proposed as a promising method for decellularisation by means of bursting the cells with high pressure and removing nuclear fragments from the tissues (Refs 54, 55). As the critical pressure (7.4 MPa) and temperature (31 °C) of CO_2_ to reach supercritical phase are relatively mild, processing of various biological materials has been reported (Refs 56, 57, 58). SC-CO_2_ shows the properties of both liquids and gases, diffuse through solids like a gas and dissolve substances like liquid allowing a low viscosity and high transport characteristics for simple and short decellularisation protocols. Additionally, SC-CO_2_ has been reported to enhance protein stability (Ref. 59), which would highly contribute to maintaining the cytocompatibility. However, the addition of a polar solvent is necessary to remove charged molecules such as DNA and phospholipids (Refs 55, 60). Although SC-CO_2_ have been successfully used in the decellularisation of dense tissues such as heart, liver, cartilage and soft tissues such as adipose tissue, which is structurally similar to the brain, it has not been used for decellularisation of CNS structures. In addition to its use in decellularisation, the recent study conducted by our group has shown that supercritical fluid technology can also be used in the sterilisation of decellularised materials (Ref. 61). In this study, SC-CO_2_ with/without ethanol, ultraviolet irradiation and ethylene oxide sterilisation techniques were applied to sodium dodecyl sulphate (SDS)-decellularised sheep brain cortical slices to determine the most effective and mild sterilisation technique. The results showed that SC-CO_2_ entrained with 6% ethanol sterilised decellularised brain tissues by yielding better results with respect to preservation of ECM proteins (Ref. 61).

The majority of the physical methods are frequently applied in combination with chemical or enzymatic methods as complete removal of cellular debris from tissue is not achieved. Until now, several types of chemicals have been used in decellularisation, including acids, bases, hypotonic and hypertonic solutions, detergents, alcohols and other solvents (Table 2). Detergents are amphipathic molecules, containing a hydrophilic head group and a hydrophobic carbon tail. Detergents effectively penetrate the phospholipid bilayer by spontaneous formation of spherical micellar structures in aqueous solutions under optimised temperature and concentration. Overall, detergents used for decellularisation can be divided into three groups: non-ionic (i.e., Triton X-100), ionic (i.e., SDS) and zwitterionic (i.e., 3-([3-colamidopropyl] dimethylammonio)-1-propanesulfonate (CHAPS)). SDS, as the most effective agent, dissolves cell and nuclear membranes by breaking down lipid–lipid and lipid–protein interactions. However, it also leads to the denaturation of structural and functional proteins by disrupting protein–protein interactions and remove GAGs and growth factors from the ECM (Refs 62, 63). Compared to ionic detergents, zwitterionic detergents exhibit better ECM preservation (Refs 64, 65). Triton-X-100 breaks down DNA–protein, lipid–lipid and lipid–protein interactions and may cause the reduction of GAG content in dECM while preserving protein–protein interactions (Ref. 66). In recent years, detergents have been used in combination to investigate their synergistic effects on decellularisation efficiency. The combined detergent decellularisation protocol showed effective decellularisation with minimal effects on tissue strength and structure in comparison to using detergents individually, which lead to detrimental effects on tissue structure and integrity or ineffective decellularisation (Refs 67, 68).

**Table 2.** A summary of chemical methods

Agents/techniques		Mode of action	Advantage/disadvantage	Tissue/organ	Ref.
Surfactant-based	SDS	Solubilise nuclear and cellular membrane Break-down the lipid–lipid, lipid–protein and protein–protein interactions	Effective decellularisation Alters microstructure Can remove growth factor and GAG from dECM	Aortic valve; ovary	(Refs 62, 63)
	Triton-X-100	Break-down the lipid–lipid, lipid–protein interactions	Can remove GAG from dECM	Anterior cruciate ligament	(Ref. 66)
	CHAPS	Acts both an ionic and non-ionic detergent	Maintains structural ECM proteins Maintains ultrastructure	Lung	(Refs 64, 65)
	The combined detergent		Minimal detrimental effects on tissue structure and integrity	Liver, pericardium	(Refs 67, 68)
Acid and bases	Peracetic acid, calcium hydroxide, sodium hydroxide, etc.	Decomposes nucleic acids Catalyses hydrolytic degradation of cellular components	Minimal negative impact on the ECM Alters mechanical properties of dECM	Meniscus	(Ref. 70)
Hypertonic, hypotonic solutions		Disrupt cell membrane by inducing osmotic shock Breaks down DNA–protein interactions	Several cycles are needed	Myocardium	(Ref. 123)
Alcohols	Isopropanol, ethanol, methanol, etc.	Lyses cells through dehydration	Tissue fixation Alters mechanical properties of dECM Rapid lipidisation	Adipose	(Refs 124, 125)

Acids and bases decellularise tissues by catalysing hydrolytic degradation of cellular components and decomposing nucleic acids. Bases are particularly used to remove hair from the dermis sample before starting the process of decellularisation (Ref. 69). But the pretreatment is advised to be carried out with caution as the mechanical properties of the ECM are significantly altered. Peracetic acid is widely preferred to remove residual nucleic acids with the minimal negative impact on the ECM (Ref. 70). As another group of treatment agents, hypertonic and hypotonic solutions induce osmotic shock in tissues, leading to the breakdown of the cell membrane. To achieve the maximum osmotic effect, tissues are usually immersed alternately in hyper and hypotonic solutions for several cycles (Ref. 41). Alcohols lyse cells by causing dehydration and breaking down the cell membrane. However, alcohols (i.e., ethanol) are used as tissue fixatives in histology, which might result in poor decellularisation. Furthermore, alcohols might alter the mechanical properties of the ECM by causing excessive shrinkage and hardening (Ref. 61).

Various enzymatic (i.e., nucleases, proteases, collagenases, lipases, dispases) and non-enzymatic (i.e., chelating agents) agents are used in biological methods (Table 3). To complete tissue decellularisation by eliminating cell remnants of the ECM, enzymatic agents are often applied after physical and chemical treatments (Refs 37, 71). Among these agents, deoxyribonuclease and ribonuclease degrade DNA and RNA, respectively.

**Table 3.** A summary of biological methods

Agents/techniques		Mode of action	Advantage/disadvantage	Tissue/organ	Ref.
Enzymes	DNase/RNase	Degrade DNA and RNA	Effectively remove residual nucleic acids from dECM	Brain, myocardial tissue	(Refs 37, 123)
	Trypsin	Cleaves the interactions between proteins	Accelerates the decellularisation process Can remove GAGs Can disrupt the fibrosis	Annulus fibrosis, cartilage	(Refs 72, 73)
Non-enzymatic agents	Chelators	Breaks down cell-matrix protein adhesions	Usually used in combination with enzymes (i.e., trypsin)	Small intestine	(Ref. 74)

Proteases such as trypsin cleave the interactions between proteins by hydrolysing peptide bonds and accelerate the decellularisation process (Ref. 72). On the other hand, it can cause a change in the quantification of GAGs to disrupt ECM structure (Ref. 73). Chelating agents such as ethylenediaminetetraacetic acid are often coupled with enzymes (i.e., trypsin) and used in breaking down cell–ECM protein adhesions (Ref. 74).

**Fig. 2.**
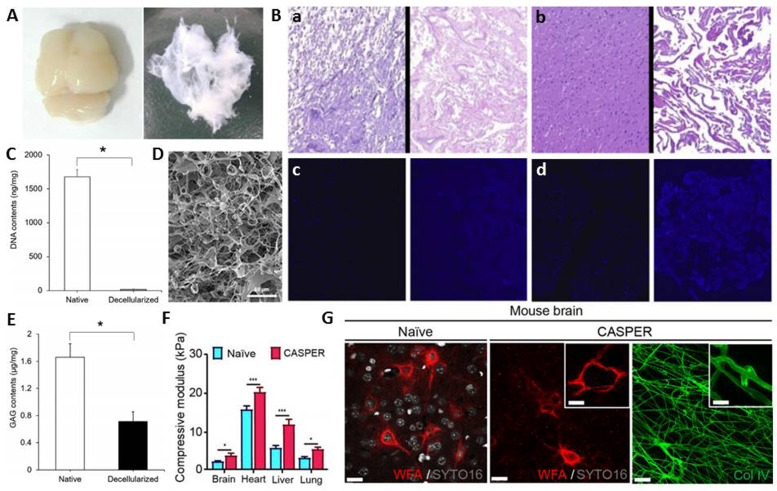
(**A)** Image of native brain (left) and dbECM (right) (Reprinted with permission from Lin et al., 2017. Copyright (2016) Elsevier) (Ref. 78). **(B)** (a) After H&E staining, cell nuclei are visible in (left) native spinal cord but not in (right) dscECM, (b) cell nuclei are visible in (left) native brain but not in (right) dbECM, (c) and (d) DAPI staining shows similar results (Reprinted with permission from Crapo et al., 2012. Copyright (2012) Elsevier) (Ref. 42). **(C)** DNA quantification shows lower concentrations of DNA in dbECM (17 ± 4 ng DNA/mg) compared to native brain (1675 ± 105 ng DNA/mg) (Reprinted with permission from Hong et al., 2020. Copyright (2019) Elsevier) (Ref. 80). **(D)** Scanning electron microscopy image (SEM) of dbECM hydrogel (Reprinted with permission from Koh et al., 2018. Copyright (2018) Springer Nature) (Ref. 82). **(E)** GAG quantification shows lower concentrations of GAG in dbECM (0.7 ± 0.1 *μ*g GAGs/mg) compared to native brain (1.6 ± 0.2 *μ*g GAGs/mg) (Reprinted with permission from Hong et al., 2020. Copyright (2019) Elsevier) (Ref. 80). **(F)** Comparison of mechanical properties of CASPERised brains and native brain (Reprinted with permission from Lee et al., 2019. Copyright (2019) American Chemical Society) (Ref. 83). **(G)** Immunofluorescence staining of the CASPERised brain with wisteria floribunda agglutinin (WFA, red) and collagen type IV (green) (Reprinted with permission from Lee et al., 2019. Copyright (2019) Elsevier) (Ref. 83).

Despite, the significance of brain–ECM microenvironment for the development of *in vitro* disease models, mostly synthetic or gelatinous protein mixtures secreted by Engelbreth-Holm-Swarm mouse sarcoma cells have been utilised. Such models have limited utility and lack physiological relevancy. Decellularisation of CNS structures can address this challenge by providing high biomimicry. In general, multiple freeze–thaw cycles are used as a pretreatment (Refs 37, 75) of CNS tissues, which is usually followed by treatment with chemical and biological agents in a mechanical agitator (Refs 25, 76, 77, 78) (×Fig. 2A). Most decellularisation studies reported significant reductions in DNA quantity and no residual nuclei in H&E and DAPI images in dbECM and dscECM (Refs 42, 79, 80, 81) (Fig. 2B and C). Human brain tissues decellularised by Triton-X-100 have been shown to exhibit porous, fibrous structures (Fig. 2D) and contain ECM components such as collagen, hyaluronic acid and GAGs (Ref. 82).

It is worth to note that brain and spinal cord are the most fragile parts of the body and contain lower amounts of fibrous protein compared to non-neuronal tissues. Thus, the number of freeze–thaw cycles have to be optimised which might otherwise cause disruption of the ECM ultrastructure. As such, chemical and biological agents may breakdown the lipid–protein and protein–protein interactions, resulting in the removal of proteins and GAGs (Refs 80, 81) (Fig. 2E). Consequently, the presence of low levels of structural and adhesion proteins as well as GAGs adversely affects cell adhesion and leads to loss of physical strength of dECM. Optimisation of consecutive decellularisation methods is required to elicit process conditions maximizing the concentrations of specific ECM components. The content of dECM is suggested to be improved by addition of various biomaterials such as collagen I, fibronectin, or synthetic polymers or the employment of crosslinking agents. However, both cases have disadvantages of increase in processing time and costs as well as an alteration in biocompatibility of the material which might alter cell-ECM interactions. Therefore, there is a need to develop innovative methods that allow efficacious decellularisation of CNS structures by avoiding excessive physical applications or long-term treatment with chemical and biological agents at high concentrations.

For instance, more recently, a novel decellularisation procedure called CASPER (Clinically and Experimentally Applicable Acellular Tissue Scaffold Production for Tissue Engineering and Regenerative Medicine) is developed, which is inspired by tissue clearing techniques such as CLARITY and ACT to prevent excessive destructive effects of chemical decellularisation methods (Ref. 83). Briefly, the procedure consists of three main steps, including the immersion of unfixed organs in a hydrogel monomer solution containing acrylamide and bis-acrylamide, the polymerisation of the hydrogel monomer by increasing the temperature to 37 °C, and subsequently the incubation of samples with a 2–4% SDS solution to remove cells from the organs. The inclusion of the infusion and hydrogel polymerisation steps before the chemical treatment with SDS prevented excessive damage to the ECM and produced a highly porous CASPERised mouse brain matrix with an intact structure (Fig. 2F). In addition, immunofluorescence staining showed that lectin (wisteria floribunda agglutinin) and collagen structures were preserved during the process of decellularisation (Fig. 2G).

## Post-processing of decellularised tissues

Almost every tissue and organ such as small intestine (Refs 84, 85), oesophagus (Refs 86, 87), liver (Refs 88, 89), lung (Refs 90, 91), dermis (Refs 92, 93), etc., have been decellularised until now. Historically, dECMs have been first used as patches/sheets or whole organs without a further breakdown in the dECM microstructure (Refs 94, 95, 96). Although many solid dECMs products have received FDA approval and demonstrated promising results in preclinical and clinical studies (Ref. 97), the majority have limitations concerning clinical utility. One of the important reasons is the implantation of scaffolds into tissue through invasive surgeries. This procedure may cause trauma in the area of interest and delay healing. Furthermore, solid scaffolds are not able to completely fill the defect areas. To facilitate the applicability of dECMs and widen the spectrum of the applications, injectable soluble dECMs have been developed by processing solid dECMs (Ref. 98). Injectable dECM are generally prepared through two approaches. The first approach is based on the generation of soluble dECM powder form, where lyophilised dECM is mechanically ground into a fine powder and then solubilised form is utilised. The second approach is based on the generation of dECM hydrogel form. For that purpose, the particulate ECM is digested by the acid-pepsin solution and then re-equilibrated to neutral pH and salt. Solubilised ECM can self-assemble into a nanofibrous hydrogel at physiological temperature (37 °C). It is worth to mention that during the solubilisation process, pepsin can disrupt the collagen fibre and completely change the composition, structural integrity and mechanical properties (Refs 99, 100). ECM hydrogels can be used in many applications such as regenerative medicine, tissue engineering, 2D and 3D cell cultures, bioprinting, and electrospinning. In addition, ECM hydrogels serve as attractive substrates for 3D organoid culture to promote proliferation and differentiation of SCs as the *in vivo* microenvironment of SC niche can be accurately recapitulated (Refs 101, 102).

## Innovative applications of decellularised CNS structures for regenerative medicine

**Fig. 3.**
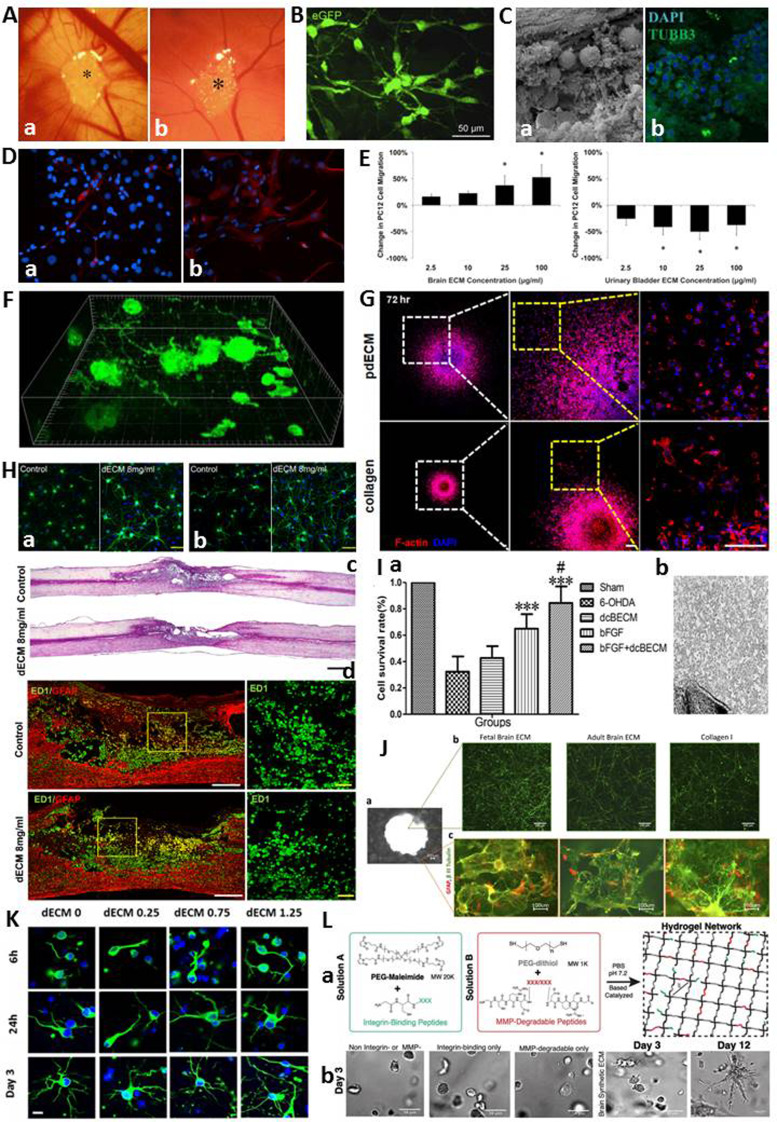
**(A)** Macroscobic images of the dbECM are surrounded by allantoic vessels after 12 days of incubation (Reprinted with permission from Ribatti et al., 2003. Copyright (2003) Elsevier) (Ref. 71). **(B)** Confocal imaging of a 3D maze-like cellular structure in 3D dbECM section (Reprinted with permission from deWaele., 2015. Copyright (2014) Elsevier) (Ref. 103). **(C)** (a) Internal region of dbECM 24 h after recellularisation with Neuro2a cell. Scale = 50 *μ*m. (b) Neuro2a cells cultures inside dbECM for 72 h. Marker for mature neuron (Tubulin beta 3; TUBB3; green) and nuclear staining (DAPI; blue) (Reprinted with permission from Granato et al., 2020. Copyright (2019) Elsevier) (Ref. 104). **(D)** (a) Immunofluorescence staining for III-tubulin (b) and GFAP (Reprinted with permission from Zhu et al., 2015. Copyright (2015) Elsevier) (Ref. 107). **(E)** (a) PC12 cell migration rate in dbECM (b) and dubECM (Reprinted with permission from Crapo et al., 2012. Copyright (2012) Elsevier) (Ref. 42). **(F)** N1E-115 cell extension following 7 days culture in B-ECM in 3D cube (Reprinted with permission from Medberry et al., 2013. Copyright (2012) Elsevier) (Ref. 77). **(G)** Filamentous actin (F-actin) staining of pdGCs in pdECM and collagen matrices (Reprinted with permission from Koh et al., 2018. Copyright (2018) Springer Nature) (Ref. 82). **(H)** (a) Immunocytochemical images of hippocampal (green) and (b) cortical neurons (green) in the hydrogels, stained for Tuj-1 for neurites and DAPI for nuclei. (c) H&E staining of the injured spinal cord tissue sample (control) and after implantation for 8 weeks with dbECM hydrogels. (d) H&E staining shows the change in cavity size (Reprinted with permission Hong et al., 2020. Copyright (2019) Elsevier) (Ref. 80). **(I)** (a) The cell survival rate under the treatment of dbECM, bFGF and bFGF + dbECM. (b) The biocompatibility of dbECM with bFGF in rat brain after implantation for 15 days (Reprinted with permission from Lin et al., 2017. Copyright (2016) Elsevier) (Ref. 78). **(J)** Growth of primary cortical rat neurons in 3D hydrogel shown by *β*-III tubulin staining for neurons (Reprinted with permission from Sood et al., 2016. Copyright (2015) American Chemical Society) (Ref. 110). **(K)** Immunostaining of encapsulated primary cortical neurons in 3D scaffolds (*β*-tubulin staining (green) for neurites and DAPI staining (blue) for cell nuclei) (Reprinted with permission from Seo et al., 2020. Copyright (2019) American Chemical Society) (Ref. 81). **(L)** (a) Schematic representation of a Michael-addition reaction used to form a hydrogel network. (b) Images of human primary astrocyte morphology in the different hydrogel conditions (Reprinted with permission from Galarza et al., 2020. Copyright (2020) WILEY) (Ref. 111).

The role of dECM in tissue regeneration and repair process is of prime importance. In this context, it is possible to use biocompatible and biodegradable solid or soluble dECM formulations. One of the first studies on brain decellularisation focused on implantation of decellularised scaffolds to chick embryo chorioallantoic membrane to examine the angiogenic response induced by dbECM scaffolds. After implantation, the results showed that dbECM scaffolds induced a strong angiogenic response, comparable to fibroblast growth factor-2 (FGF-2), an angiogenic cytokine (Ref. 71) (Fig. 3A). Different studies also showed that dbECM scaffolds support sustained *ex vivo* growth of different types of cells such as murine neural SCs (NSCs) (Ref. 103) (Fig. 3B) and Neuro2a cells (Ref. 104) (Fig. 3C). In addition to the dbECM, decellularised spinal cord and cerebellar scaffolds have also used in tissue engineering applications. For instance, decellularised scaffolds from spinal cord with preserved 3D structure and composition were implanted in rats and the density of CD4+ and CD8+ cells that infiltrated the scaffolds after implantation was examined by immunohistochemical analysis. The results showed that dscECM was invaded by CD4+ and CD8+ cells to a much lesser extent than an allologous spinal cord graft, possibly due to an effective decellularisation protocol allowing the removal of myelin and cells that might have initiated an immune response after implantation (Ref. 105). To evaluate the effect of the dscECM as an allograft on functional improvement in spinal cord injury-induced adult rats, dscECM scaffold was implanted alone and seeded with human umbilical cord (UC) blood-derived MSCs (hUCB-MSCs). The behavioural analysis indicated that, dscECM scaffold and dscECM scaffold + hUCB-MSCs provided significant locomotor recovery improvement. The scaffold supported hUCB-MSCs proliferation, as well as the migration of host neural cells into the graft. Furthermore, it is observed that myelinated axons could grow into the graft, promoting axonal regeneration at lesion sites (Ref. 106). More recently, a decellularised cerebellum has been produced exhibiting *in vitro* and *in vivo* biocompatibilities. As cerebellum-derived ECM maintained a native microenvironment, seeded NSCs have been able to differentiate into neurons and astrocytes expressing cell-specific proteins, III-tubulin and glial fibrillary acidic protein, respectively (Fig. 3D). To assess immunogenicity, the dscECM scaffold was implanted subcutaneously on the dorsal side and intracranially into the frontal lobes of rats. After 4 weeks of implantation, low numbers of CD4+ or CD8+ monocytes were observed in all subgroups (Ref. 107).

To facilitate applicability of dECMs and widen the range of their applications in CNS studies, a method was developed to process decellularised porcine brain into a solubilised form (Ref. 76). In this study, the hydrogel was used as a coating material for culture of neurons, where human iPSC-derived neurons plated on the hydrogel expressed neuronal markers and showed neuronal morphology. Additionally, the solubilised brain matrix was reported to self-assemble into nanofibrous hydrogel upon injection *in vivo*. This milestone study demonstrated the feasibility of the solubilised brain matrix for cell culture studies as a coating material and tissue engineering applications as a hydrogel scaffold. To prepare the ideal ECM hydrogel form for CNS repair, both neuronal and non-neuronal tissues were prepared and evaluated *in vitro* and/or *in vivo* (Refs 28, 42, 77). In a study, the addition of solubilised optic nerve, brain and spinal cord ECMs to PC12 neuronal cultures resulted in induced proliferation, migration and differentiation *in vitro* (Ref. 42). On the contrary, solubilised urinary bladder ECM (non-CNS ECM) showed an inhibitory effect on PC12 cell migration rather than acting as a chemoattractant over the same concentration (Ref. 42) (Fig. 3E). Moreover, CNS ECM has been reported to induce differentiation from NSCs into neurons expressing bIII-tubulin in two-dimensional culture, whereas neurite extension in three-dimensional culture (Ref. 28). In another study, both CNS and non-CNS ECM (urinary bladder) hydrogels have been reported to increase the number of N1E-115 cells expressing neurites; however, only brain ECM increased neurite length (Ref. 77) (Fig. 3F). More recently, dbECM, dscECM and decellularised UC hydrogels have been shown to promote the migration of human MSCs and differentiation of NSCs, as well as axonal outgrowth *in vitro* (Ref. 108). However, only ducECM hydrogel could significantly improve the proliferation of tissue-specific UC-derived MSCs when compared with CNS ECMs (Ref. 108). All these results have shown that CNS ECMs provide tissue-specific effects on cell lines. Indeed, hydrogels prepared from dbECM have shown promising results in brain tissue remodelling and repair following traumatic brain injury (Ref. 109).

Hydrogels derived from dECM have also been used in *in vitro* 3D tumour models. Such *in vitro* 3D models are of prime importance to determine the effect of tumour-specific ECM on the propagation mechanism of cancer cells. For instance, a 3D *in vitro* tumour model was developed by using a hydrogel derived from patient tissue-ECM (pdECM) to investigate the invasive characteristic of glioblastoma multiforme (GBM). In this study, the results showed that the disseminated GBM cells exhibited significantly different morphologies within the pdECM and collagen matrix (Fig. 3G) (Ref. 82).

Subsequent to injection, the host response to hydrogel is critical in terms of success or failure in tissue-repair applications. Macrophages are vital in the host response to implanted biomaterials and have been reported as predictors of downstream tissue remodelling events. For instance, one of the studies has shown that porcine bECM hydrogel expressed a predominant M2-like macrophage phenotype, which is pro-remodelling and anti-inflammatory (Ref. 112). In another study, 3D hydrogels derived from adult porcine brain dECM powder and collagen I solution have been evaluated for neurite outgrowth of cortical and hippocampal neurons and reported to promote macrophage polarisation towards M2 phenotype for *in vitro* SCI model. Also, these brain dECM hydrogels were used as injectable biomaterials for *in vivo* rat SCI model, and shown to modulate the macrophages in the injured spinal cord for neuroregeneration (Ref. 80) (Fig. 3H).

The delivery of therapeutics such as bioactive molecules, drugs and growth factors via 3D brain dECM scaffolds or brain dECM hydrogel-based nano/micro particular system is an important approach to stimulate cell-tissue growth for the regenerative medicine application and the treatment of CNS diseases. bFGF is a member of the FGFs and regulates important biological functions such as stimulating angiogenesis and neuroprotection (Ref. 113). However, bFGF has a short-half life in free form. To overcome this, CNS-derived rat brain dECM scaffold containing bFGF was used to enhance and extend the neuroprotective effect of bFGF on Parkinson's disease targeted therapy. The biocompatibility and neuroprotective effect of bFGF doped dECM were evaluated *in vitro* PC-12 cell culture model and *in vivo* rat model (Fig. 3I). *In vivo* study showed that the combination of dbECM and bFGF may be a promising and safe therapeutic strategy for Parkinson's disease as neural recovery was promoted (Ref. 78). In a similar study, bFGF-dscECM scaffold complex was encapsulated into a heparin modified poloxamer (HP) solution to prepare a temperature-sensitive hydrogel (bFGF-dscECM-HP) (Ref. 114). An *in vitro* cell survival study showed that the bFGF-dscECM-HP hydrogel promoted the proliferation of PC12 cells more in comparison to the bFGF solution. Glial scars were reported to be inhibited by bFGF-ASC-HP hydrogel and contributed to recovery through differentiation of the NSCs and regeneration of nerve axons. In another study, neurotrophin-3 that supports adhesion, proliferation and differentiation of rat BMSCs was cross-linked with dscECM to construct a sustained-release system, where 35 days of neurotrophin-3 release was achieved (Ref. 115).

Within the scope of regenerative medicine applications, 3D composite hydrogel systems are one of the most commonly used decellularised brain tissues to mimic functional, structural and biochemical properties of native brain tissue, which might be further developed for neuroregeneration. A 3D bioengineered composite hydrogel model of cortical brain tissue was developed consisting of fibrous mesh-like dECM from foetal porcine brain, collagen I solution, silk scaffold and embryonic day 18 primary neurons, to provide a framework for studying axonal ingrowth of cortical neurons *in vitro* (Ref. 110) (Fig. 3J). In a similar study, a 3D composite hydrogel mixing porcine brain dECM and collagen I, with/without encapsulated primary embryonic cortical neurons was reported to recapitulate the brain tissue microenvironment through increased neuronal differentiation/outgrowth and neuron-to-brain dECM interactions. Subsequent to microfibril alignment by stretching and releasing of the hydrogel-based chip to obtain anisotropically organised brain dECM, neurons incubated in this 3D platform were reported to exhibit enhanced neurite outgrowth and development compared to only collagen gels (Ref. 81) (Fig. 3K). In another study, a spinal cord anatomy-inspired hyaluronic acid-ECM composite hydrogel was formulated with the core ECM derived from grey matter regions of the brain, whereas the shell from white matter regions (Ref. 25) and primary mouse ESCs were encapsulated in the hydrogel. The ESCs have been reported to survive, spread in the scaffold and differentiate into neuronal lineages, expressing nestin. To enhance the mechanical strength of ECM scaffolds and prevent rapid degradation by host enzymes such as collagenase and connective tissue proteases after a certain period of time, crosslinking has been applied in various studies. For instance, genipin-crosslinked rat dscECM enabled the structural integrity of the scaffolds to be preserved for 14 days *in vitro* (Ref. 116). In another study, a rat brain dECM incorporated genipin-crosslinked gelatin electrospun scaffold was developed to provide a suitable microenvironment for cell adhesion, proliferation, survival and differentiation potential towards neural precursor cells for nervous tissue regeneration (Ref. 37). As a carboxyl- and amine-reactive cross-linker, EDC was also utilised to enhance the properties of dscECMs and provide adhesion and differentiation of rat BMSCs into neuron-like cells (Ref. 117). With an innovative approach, a 3D synthetic brain hydrogel containing human brain dECM peptide mixture, enriched with crosslinked hyaluronic acid by linear PEG-dithiol was developed and functionalised with brain-specific integrin binding/matrix metalloprotease degradable peptide cocktail, which was shown to control astrocyte star-shaped morphologies and maintenance of astrocyte quiescence compared to collagen hydrogels (Ref. 111) (Fig. 3L).

The microfluidic-based organ-on-a-chip systems that mimic *in vivo* microenvironment by incorporating dECM may be useful to understand the extrinsic factors involved in the regulation of cell fate and function, and also for fast and cost-effective personalised drug screening studies (Ref. 118). With this regard, a patient-specific *ex vivo* glioblastoma-on-a-chip model was developed with a highly biomimetic ecosystem, which consisted of bioprinted patient-derived tumour cells and vascular endothelial cells with dbECM bioink, and yielded important cues for recapitulating the pathological features of glioblastoma, thus allowing for patient-specific drug susceptibility to be identified (Ref. 119). The results suggested that tumour-on-a-chip model containing dbECM bioink was an option for modelling personalised cancer treatments and for drug screening to guide clinical decisions that may overcome refractory cancers. In another study, human brain dbECM-based cell culture system was used to facilitate plasmid-transfection-based direct conversion of primary mouse embryonic fibroblasts into induced neuronal cells. The results showed that microfluidic system combined with the 3D dbECM hydrogel was able to closely mimic *in vivo* conditions for neuronal reprogramming (Ref. 120). As for drug screening, a 3D biomimetic macro-porous scaffold was fabricated by incorporating hyaluronic acid, porcine brain ECM and growth factors to create a patient-derived xenograft model by using primary glioma SCs (GSCs). The platform has been reported to regenerate primary gliomas and used for screening novel siRNA nanotherapeutics to inhibit the tumorigenic potential of GSCs with current clinical drug, temozolomide and an anticancer phytochemical, nanocurcumin as a control (Ref. 121). For potential drug screening/toxicity assays, human brain organoids are clinically relevant and scalable models. In a recent study, porcine brain dECM hydrogels provided similar gene expression and differentiation of ESCs similar to those grown in matrigel outcomes and suggested as an alternative scaffold for human cerebral organoid formation (Ref. 122).

## Challenges and future prospects

The combination of decellularisation techniques with complex biology has enabled the development of advanced applications in regenerative medicine and neural tissue engineering. Current decellularisation methods have been successful to a certain extend. For instance, chemical and biological methods might lead to degradation of organ-specific ECM components, whereas physical methods such as supercritical CO_2_ and electroporation can yield insufficient and non-homogeneous decellularisation when applied alone. Thus, a combination of different techniques can be consecutively applied to preserve the ECM niche. Along with the decellularisation method, the sterilisation method also affects the biochemical composition, ultrastructure and mechanical properties of decellularised matrix-based biomaterials. Thus, comprehensive investigation of appropriate sterilisation techniques for dbECM and dsECM-based biomaterials is required. Studies conducted to date have shown that dbECM and dscECM-based biomaterials are biocompatible, angiogenic, able to regulate various cell behaviours such as differentiation and migration. Furthermore, these biomaterials provide *in vitro* and *in vivo* controlled release of growth factors, promote neural recovery and neuroprotection by exhibiting low immunogenicity subsequent to *in vivo* implantation. In the next years, the use of dbECM and dscECM-based hydrogels with 3D printing technology may accelerate the development of therapeutic solutions. Although the low viscosity and poor mechanical properties of dbECM and dscECM-based hydrogels limit their use in 3D bioprinting technology, the combination with well-known synthetic or natural hydrogels may enable the printing. Given the significance of brain- and spinal cord-ECM microenvironment, development of therapeutic solutions will be expedited by post-processing of dECM and eventually, new opportunities are envisaged for the treatment of neuronal diseases and injuries.
